# Supplementary material for: Evolutionary insights into 3D genome organization and epigenetic landscape of Vigna mungo
Source: Life Sci Alliance. 2023 Nov 3;7(1):e202302074. doi: 10.26508/lsa.202302074 (PMC10624639; doi:10.26508/lsa.202302074)
Supplement: Supplementary file 1 [file LSA-2023-02074_TableS1.docx]

|  | **SMRT1** | **SMRT2** | **SMRT3** | **SMRT4** |
| --- | --- | --- | --- | --- |
| **Polymerase Read Bases** | **13,836,681,578** | **13,027,951,507** | **15,040,207,506** | **14,273,995,410** |
| **Polymerase Reads** | **532,584** | **505,319** | **563,995** | **559,919** |
| **Polymerase Read Length (mean)** | **25,980** | **25,782** | **26,667** | **25,493** |
| **Polymerase Read N50** | **54,567** | **52,484** | **55,957** | **50,954** |
| **Subread Length (mean)** | **13,727** | **14,000** | **13,987** | **13,761** |
| **Subread N50** | **18,374** | **18,684** | **18,685** | **18,755** |
| **Insert Length (mean)** | **13,810** | **14,115** | **14,106** | **14,072** |
| **Insert N50** | **19,067** | **19,396** | **19,496** | **19,636** |

Supple Table 1: PacBio reads summary
