# Supplementary material for: Evolutionary insights into 3D genome organization and epigenetic landscape of Vigna mungo
Source: Life Sci Alliance. 2023 Nov 3;7(1):e202302074. doi: 10.26508/lsa.202302074 (PMC10624639; doi:10.26508/lsa.202302074)
Supplement: Supplementary file 2 [file LSA-2023-02074_TableS2.docx]

Supple table 2: HiC Reads summary

| Sequenced Read Pairs | 419,616,668 |
| --- | --- |
| Ligation Motif Present | 282,676,390 |
| Alignable (Normal + Chimeric Paired) | 371,175,908 |
| Unique Reads | 279,821,856 |
| Hi-C Contacts | 169,800,908 |
| Inter-chromosomal | 73,711,869 |
| Intra-chromosomal | 96,089,039 |
