# Supplementary material for: Evolutionary insights into 3D genome organization and epigenetic landscape of Vigna mungo
Source: Life Sci Alliance. 2023 Nov 3;7(1):e202302074. doi: 10.26508/lsa.202302074 (PMC10624639; doi:10.26508/lsa.202302074)
Supplement: Supplementary file 3 [file LSA-2023-02074_TableS3.docx]

Supple table 3: RNA-seq read alignments summary

| Sample Name | Total number of reads | Alignment percentage |
| --- | --- | --- |
| VM-20D-Leaf-Replicate1 | 40038759 | 94.51% |
| VM-20D-Leaf-Replicate2 | 35858930 | 93.90% |
| VM-20D-root-Replicate1 | 52177409 | 90.48% |
| VM-20D-root-Replicate2 | 44200264 | 92.51% |
| VM-45D-leaf-Replicate1 | 39940094 | 91.20% |
| VM-45D-leaf-Replicate2 | 42351872 | 78.8% |
| VM-45D-root-Replicate1 | 21532844 | 92.86% |
| VM-45D-root-Replicate2 | 40276863 | 91.06% |
| VM-flower-Replicate1 | 46053074 | 97.65% |
| VM-flower-Replicate2 | 41583048 | 92.00% |
| VM-young pod-Replicate1 | 41263861 | 93.56% |
| VM-young pod-Replicate2 | 41798092 | 93.33% |
