# Supplementary material for: Evolutionary insights into 3D genome organization and epigenetic landscape of Vigna mungo
Source: Life Sci Alliance. 2023 Nov 3;7(1):e202302074. doi: 10.26508/lsa.202302074 (PMC10624639; doi:10.26508/lsa.202302074)
Supplement: Supplementary file 4 [file LSA-2023-02074_TableS4.docx]

Supple table 4: BUSCO genome completeness analysis

| BUSCO category | Number |
| --- | --- |
| Complete | 1558 (96.5%) |
| Complete and single-copies | 1509 (93.5%) |
| Complete and duplicated | 49 (3.0%) |
| Fragmented | 15 (0.9%) |
| Missing | 41 (2.6%) |
