# Supplementary material for: Evolutionary insights into 3D genome organization and epigenetic landscape of Vigna mungo
Source: Life Sci Alliance. 2023 Nov 3;7(1):e202302074. doi: 10.26508/lsa.202302074 (PMC10624639; doi:10.26508/lsa.202302074)
Supplement: Supplementary file 5 [file LSA-2023-02074_TableS5.docx]

Supple table: 5 Assembly stats of *V. mungo* assembly (Pootakham et al. 2021)

| Assembly size | 498 Mb |
| --- | --- |
| Chromosome number (2n) | 11 |
| No. of super scaffolds | 9224 |
| Super scaffold N50 | 43.1 Mb |
| No. of Ns (Gap) | 34.7 Mb |
| GC content | 33.6 % |
| No. of Genes | 32729 |
| Mean gene length | 3123 bp |
