# Supplementary material for: Evolutionary insights into 3D genome organization and epigenetic landscape of Vigna mungo
Source: Life Sci Alliance. 2023 Nov 3;7(1):e202302074. doi: 10.26508/lsa.202302074 (PMC10624639; doi:10.26508/lsa.202302074)
Supplement: Supplementary file 6 [file LSA-2023-02074_TableS6.docx]

Supple table 6: LTR proportion

| **Species** | **Class** | **Subclass** | **Proportion of genome (bp)** | **Percentage of genome** |
| --- | --- | --- | --- | --- |
| V. angularis | LTR | Copia | 36537837 | 8.159291473 |
|  |  | Gypsy | 85599834 | 19.1153624 |
|  |  | unknown | 20313985 | 4.536331053 |
| V. unguiculata | LTR | Copia | 54773511 | 10.55540497 |
|  |  | Gypsy | 104640208 | 20.16521767 |
|  |  | unknown | 30101216 | 5.800806252 |
| V. mungo | LTR | Copia | 53633160 | 11.19042164 |
|  |  | Gypsy | 80065834 | 16.70553146 |
|  |  | unknown | 33398289 | 6.968467569 |
