# Supplementary material for: Evolutionary insights into 3D genome organization and epigenetic landscape of Vigna mungo
Source: Life Sci Alliance. 2023 Nov 3;7(1):e202302074. doi: 10.26508/lsa.202302074 (PMC10624639; doi:10.26508/lsa.202302074)
Supplement: Supplementary file 8 [file LSA-2023-02074_TableS7.docx]

| Sample | Number of Reads | Aligned reads | Mapping efficiency |
| --- | --- | --- | --- |
| Leaf_Rep1 | 52194628 | 40478253 | 69.6% |
| Leaf_Rep2 | 52827410 | 41084561 | 69.8% |
| Shoot_Rep1 | 49930029 | 37595113 | 69.1% |
| Shoot_Rep2 | 39147107 | 30198830 | 71.7% |
| Root_Rep1 | 51682003 | 32220066 | 56.0% |
| Root_Rep2 | 48462102 | 22013744 | 44.4% |

Supple table 7: BS-seq read alignment summary
